# Medial clavicle fracture with bone destruction after radical neck dissection combined with postoperative chemotherapy for secondary cervical lymph node metastasis of tongue cancer: a case report

**DOI:** 10.1007/s11282-021-00515-9

**Published:** 2021-02-12

**Authors:** Masayasu Tashiro, Tomoaki Sano, Kazutaka Sugiura, Yasuhito Minamida, Yoshihiro Abiko, Hiroki Nagayasu, Eiji Nakayama

**Affiliations:** 1 Division of Oral and Maxillofacial Radiology, Department of Human Biology and Pathophysiology, School of Dentistry, Health Sciences University of Hokkaido, 1757 Kanazawa, Ishikari-Tobetsu, Hokkaido 061-0293 Japan; 2 Division of Oral Maxillofacial Surgery, Department of Human Biology and Pathophysiology, School of Dentistry, Health Sciences University of Hokkaido, 1 1 7 5 7 Kanazawa, Ishikari-Tobetsu, Hokkaido, 061-0293, Japan; 3 Division of Oral Medicine and Pathology Department of Human Biology and Pathophysiology School of Dentistry, Health Sciences University of Hokkaido, 1 7 5 7 Kanazawa, Ishikari-Tobetsu, Hokkaido, 061-0293, Japan

**Keywords:** Clavicle fracture, Oral cancer, Radical neck dissection, Diagnostic imaging

## Abstract

**Background:**

Clavicle fractures (CF) after radical neck dissection (RND) for oral cancer are rare but are thought to occur as a result of myotonia and decreased blood supply to the muscles around the clavicle after RND. The current report presents a rare case of a non-neoplastic pathological CF after RND, and discusses the role of imaging examinations for the timely detection of CF.

**Case report:**

An 82-year-old Japanese man underwent RND followed by chemotherapy without radiotherapy for secondary metastasis of the right cervical lymph node after resection of tongue cancer. Computed tomography at 6 months after RND revealed a fracture with bone destruction in the proximal end of the right clavicle. He had no history of trauma at the site of the fracture and no symptoms. The possibility of bone metastasis of the clavicle was considered; however, the bone destruction had not advanced 6 years after the discovery of the fracture. The CF was thus finally considered to be a side effect of RND, rather than metastasis.

**Conclusion:**

CF is a rare complication following treatment for head and neck cancer but can be caused by neck dissection. Regular imaging examinations, including the clavicular region, are therefore needed before and after surgery to ensure the timely detection of CF.

## Introduction

Traumatic clavicle fractures (CF) are relatively common, accounting for 4% of adult fractures [1]. CF are generally classified as medial- or middle-third fractures, or fractures distal to the coracoclavicular ligament, according to Allman’s classification [2, 3], with relative incidences of approximately 2.8%, 69.2%, and 28%, respectively [3].

In contrast, non-traumatic fractures in patients with malignant tumors are usually caused by bone metastases, but may also rarely occur after radical neck dissection (RND). Notably, fractures of the medial end of the clavicle have been reported as a rare late complication after RND, with an incidence of approximately 0.4%–0.5% [4]. Regarding their cause, Strauss et al. reported that RND or radiotherapy caused weakening of the bone and blood supply, resulting in subsequent fracture of the clavicle [5].

We recently experienced a case of a non-neoplastic fracture in the medial third of the clavicle with bone destruction after RND combined with postoperative chemotherapy, with no radiotherapy. We, therefore, present this rare case of a non-neoplastic CF after RND and chemotherapy, and discuss the role of imaging examinations for the timely detection of CF.

## Case report

**Fig. 1:**
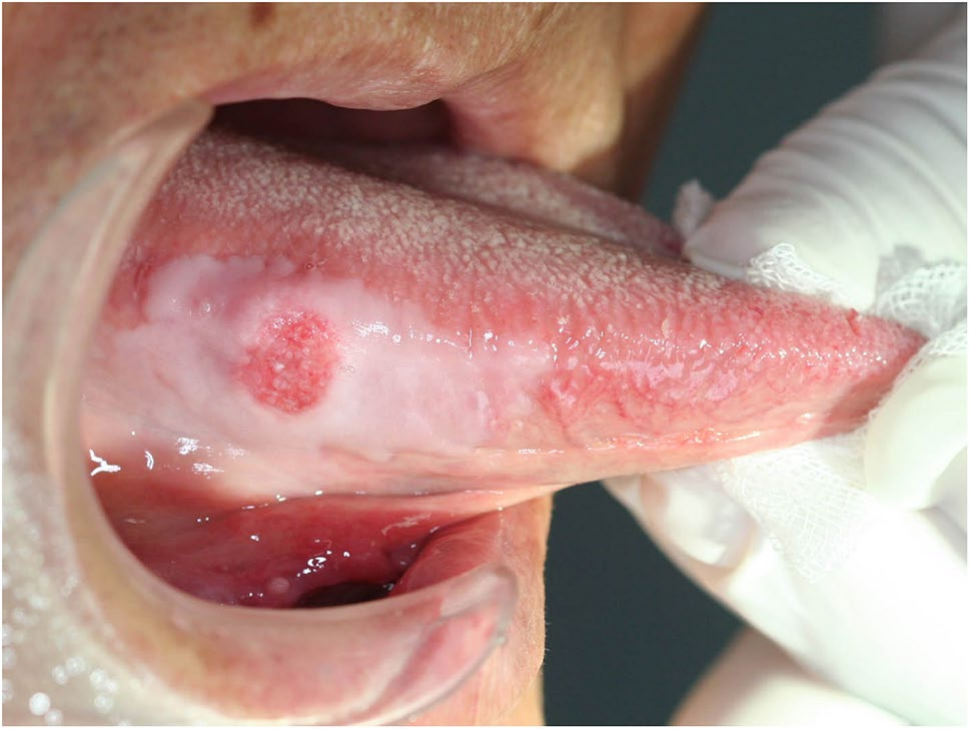
Tumor with large leukoplakia observed in the right margin of the tongue

An 81-year-old Japanese man visited our hospital with a chief complaint of pain in the right lingual margin. The patient had been aware of a mass on the right lingual surface for 4 months (Fig. 1), and finally attended our hospital because of contact pain with his dentures.

**Fig. 2:**
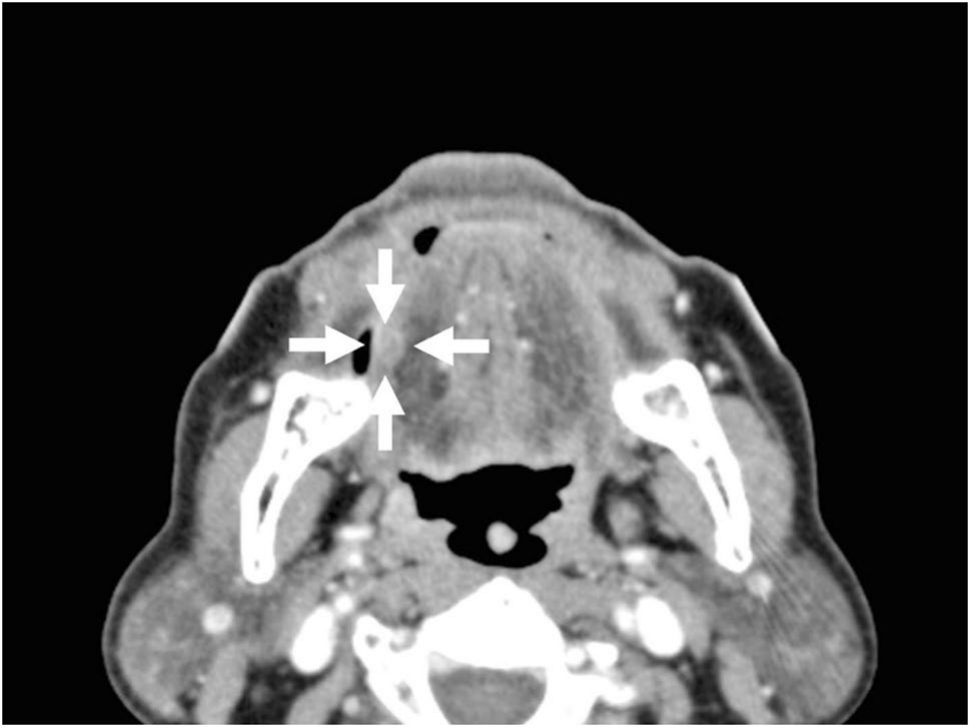
Contrast-enhanced CT revealed an enhanced soft tissue mass (arrows) at the right lingual margin, diagnosed histopathologically as squamous cell carcinoma

Contrast-enhanced computed tomography (CT) showed an enhanced soft tissue mass at the right lingual margin (Fig. 2), but no lymphadenopathies suggesting metastasis. Examination of a biopsy of the lingual mass revealed squamous cell carcinoma, with clinical TNM classification T1N0M0 (stage I). The patient was therefore hospitalized and underwent tumor resection of the right lingual site under general anesthesia on June 25, 2009.

**Fig. 3:**
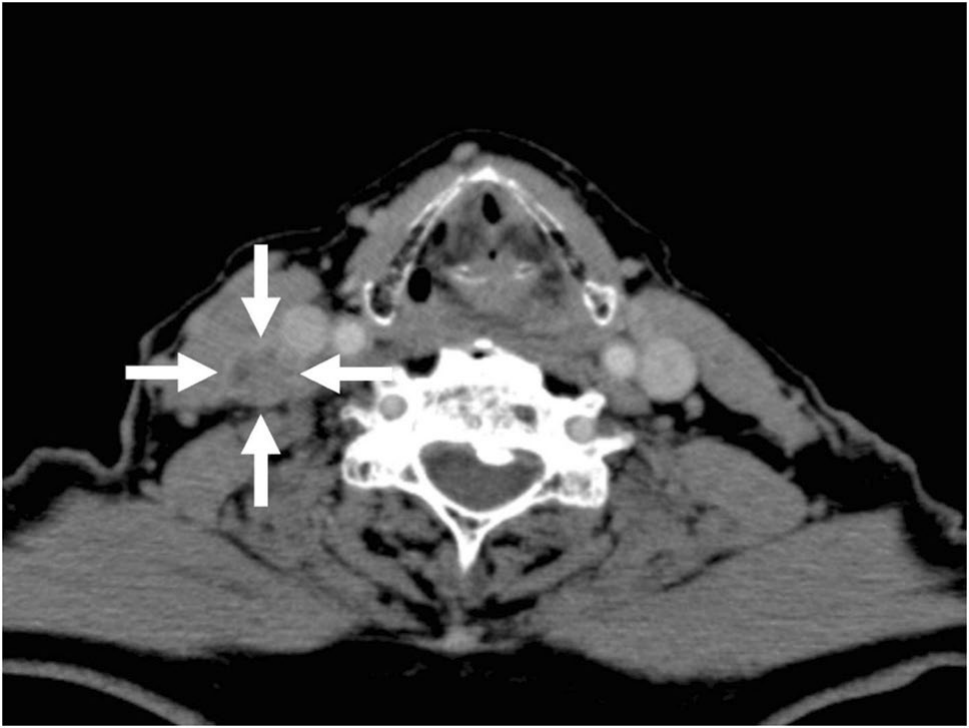
Contrast-enhanced CT 6 months after tumor resection showed a new swelling (arrows) of the right upper cervical lymph node, suggesting metastasis

Follow-up contrast-enhanced CT 8 months after tumor resection (February 15, 2010) showed a lymph node swelling at the right upper cervical region, suggesting secondary lymph node metastasis (Fig. 3). He, therefore, underwent RND under general anesthesia on March 19, 2010, according to the traditional method [6]. Postoperative adjuvant chemotherapy with fluorouracil (total dose: 8750 mg), cisplatin (total dose: 240 mg), and tegafur/gimeracil/oteracil (total dose: 1680 mg) was planned. However, because of the patient’s poor physical condition, adjuvant chemotherapy was stopped after administration of 8750 mg fluorouracil, 240 mg cisplatin, and 1560 mg of tegafur/gimeracil/oteracil.

**Fig. 4:**
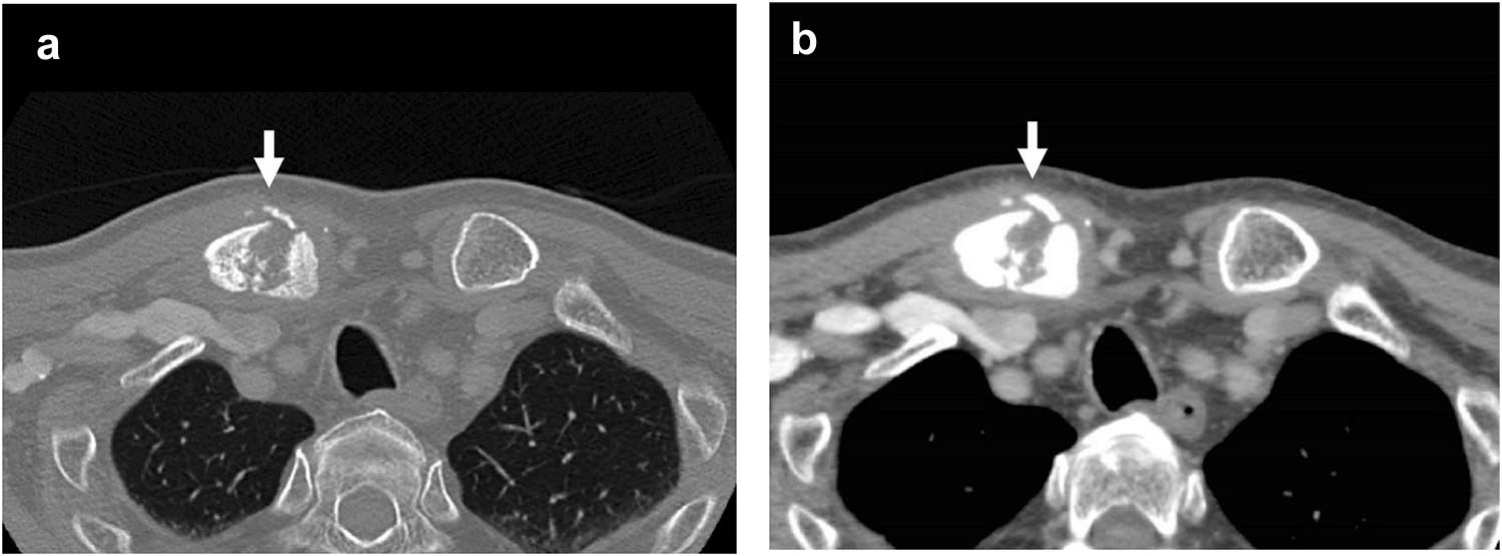
Contrast-enhanced CT (**a**: bone-window image, **b**: soft tissue-window image) on September 17, 2010, 6 months after right RND, showed a fracture with bone destruction (arrows) at the proximal end of the right clavicle

Follow-up contrast-enhanced CT 6 months after right RND (September 17, 2010) detected a fracture with bone destruction in the proximal end of the right clavicle (Fig. 4). This was regarded as a pathologic fracture, and bone metastasis from the tongue cancer was considered as a possible cause.

**Fig. 5:**
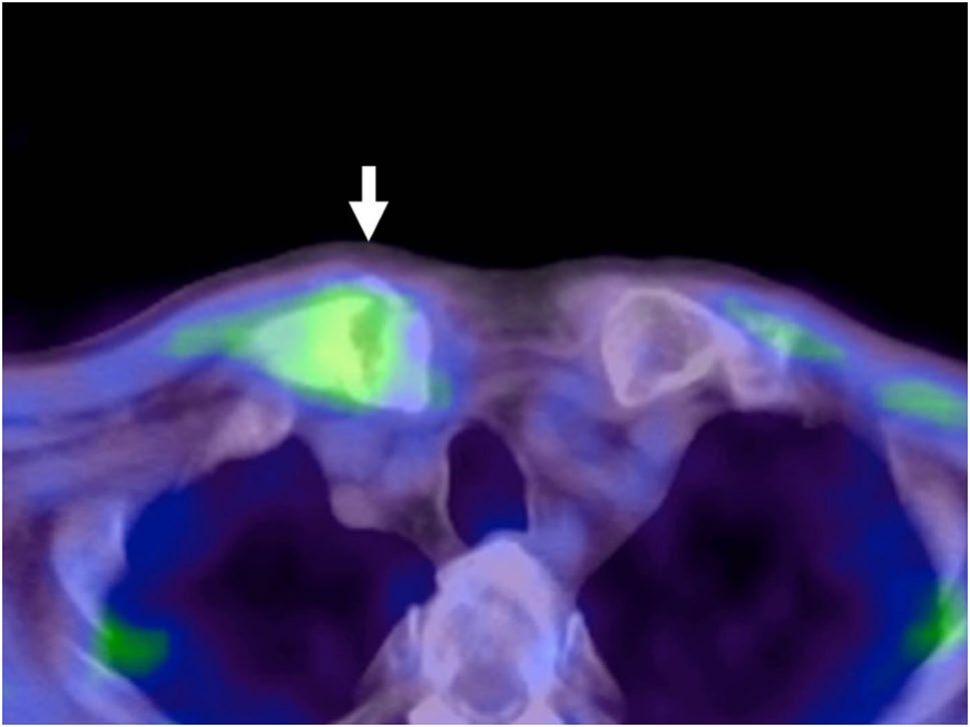
PET-CT on September 24, 2010, 1 week after detection of CF by CT, showed relatively weak accumulation (arrow) at the fractured clavicle

The patient underwent ^18^F-fluorodeoxyglcose (FDG) positron emission tomography (PET)-CT on September 24, 2010, which showed moderate and relatively diffuse ^18^F-FDG accumulation at the proximal end of the right clavicle, corresponding to the fracture site (Fig. 5). However, the level of accumulation was not very high and the maximum standardized uptake value was 3.7, and the probability of metastasis was therefore thought to be low.

Consultation 4 days after PET-CT detected swelling and slight redness of the superficial skin on the right clavicle. However, the patient reported no symptoms in the right clavicular region, and no history of trauma that might have caused a fracture of the clavicle.

**Fig. 6:**
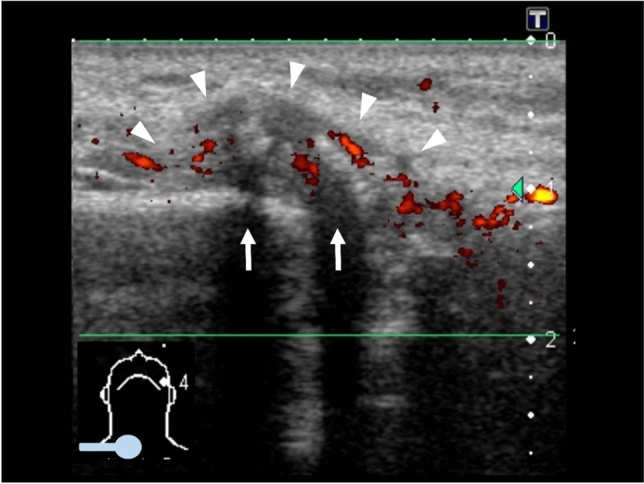
Power Doppler US on October 5, 2010 showed a hypoechoic solid mass (arrowheads) destroying the cortex of the clavicle (arrows), and with weak signals in the internal mass and surrounding blood flow

Ultrasonography (US) on October 5, 2010 showed a hypoechoic solid mass destroying the cortex of the clavicle, with weak signals in the internal mass and surrounding blood flow on power Doppler US (Fig. 6), suggesting that the CF was not caused by metastasis. Moreover, the redness disappeared 1 week after it was first noticed, although the swelling remained.

We, therefore, considered that the CF was likely to have been caused by non-neoplastic changes, rather than by bone metastasis, and accordingly adopted a watch and wait approach regarding the CF.

**Fig. 7:**
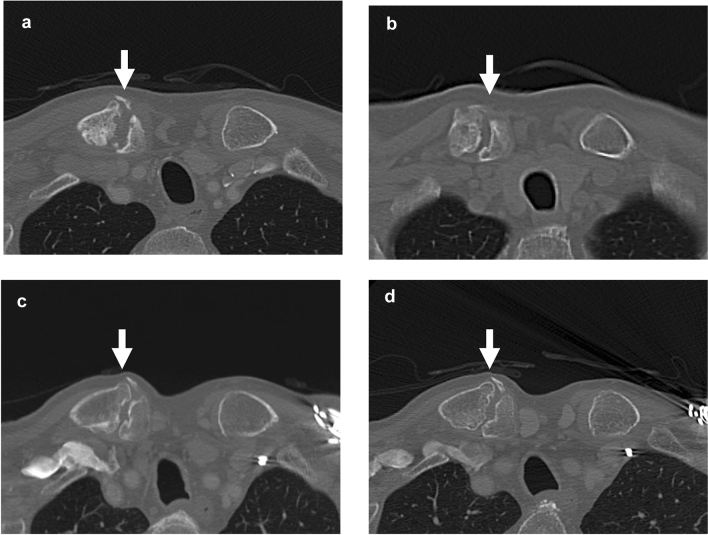
Follow-up contrast-enhanced CT examinations over the next 6 years showed gradual narrowing of the separation between the bone fragments, but no adhesion of the fragments (arrows). **a**: CT on June 20, 2011; **b**: CT on May 20, 2013, **c**: CT on November 8, 2016, **d**: CT on November 2, 2017

Four follow-up contrast-enhanced CT scans were carried out over the following 6 years, with no signs of recurrence or metastasis of the malignant tumor. Regarding the CF, the separation between the bone fragments reduced and the patient’s condition was stable without inflammation, although adhesion of the bone fragments was not achieved (Fig. 7).

In conclusion, we judged that the CF had occurred as a secondary effect of RND.

## Discussion

We experienced a patient with a medial CF following RND in 2010. We performed a total of 25 RND procedures from 2008 to 2019, and the present patient represented the only case of a CF after RND.

However, similar cases of CF after neck dissection have been reported by other researchers [4, 5, 7–15], who advocated various hypotheses regarding their causes.

Strauss et al. [5] and others [4, 7, 8, 14] considered that CF after neck dissection was a result of weakening of the bone and blood supply caused by neck dissection and/or radiotherapy. However, the current patient did not receive radiation therapy. Other reports of medial CF after neck dissection indicated that neck dissection alone might cause such fractures, with or without postoperative radiotherapy [11, 14].

Regarding the possible association between CF after neck dissection and bone destruction, Shodo et al. [13] reported a case of CF after neck dissection and postoperative radiotherapy followed by osteomyelitis with bone destruction. They suggested that osteomyelitis and abscess formation with bone destruction caused by risk factors such as radiotherapy, tracheostomy, and adjacent infection might complicate the fracture findings, such as bone destruction in clavicle stress fractures after neck dissection [13]. In both the current and Shodo et al.’s case, the fracture was accompanied by bone destruction; however, our case was not associated with infection.

Fujimoto et al.’s [14] study was also relevant to the association between CF after neck dissection and bone destruction. They assessed the CT images of nine cases of CF following neck dissection and suggested that proximal CF consistently showed an extraosseous soft-tissue mass formation without bone destruction on CT [14]. In our case, the patient had no subjective symptoms related to the fracture, despite the formation of an extraosseous soft-tissue mass, as in Fujimoto et al.’s cases. However, the fracture in the present case was accompanied by bone destruction, which was absent in Fujimoto et al.’s cases.

The pathogenesis of bone destruction in CF after neck dissection might be associated with multiple factors, and the cause for the bone destruction in the current patient has not yet been identified. However, comparing the present and previous cases of CF, our case suggests that postoperative chemotherapy after RND might contribute to bone destruction in CF, even in the absence of radiotherapy [8, 16] and infection [14] of the clavicle. The influence of chemotherapy on the occurrence of CF after neck dissection remains unknown. One study reported a possible effect of chemotherapy on bone [17], while some cases of CF following neck dissection have been reported in patients without chemotherapy [7–10], and others have been noted in cases with chemotherapy [8, 9]. Further studies are therefore needed to elucidate the influence of chemotherapy in the pathogenesis of CF after neck dissection.

In the present patient, the CF was first discovered during a follow-up imaging examination, and the CF status during the 6 months from RND to its first discovery remains unknown. We are therefore unable to explain the interaction between the fracture and the bone destruction. Future cases should thus consider the mechanism of bone destruction in CF after neck dissection.

The current case shows that CF after RND may be accompanied by bone destruction, even if the fracture is not associated with infection. Moreover, in our case, bone destruction of the CF might have progressed because the discovery of the fracture was delayed. This case highlights the need to recognize CF as one of the adverse events after neck dissection [14], and to ensure that the condition is promptly detected and treated. Regular follow-up imaging examinations, including CT, will aid the rapid discovery of CF after neck dissection for head and neck cancer [8, 13, 14].

CF is a rare complication after treatment for head and neck cancer but can be caused by neck dissection. Regular imaging examinations, including the clavicular region, should, therefore, be carried out before and after surgery to ensure the prompt detection of CF.
